# Screening of *Hibiscus* and *Cinnamomum* Plants and Identification of Major Phytometabolites in Potential Plant Extracts Responsible for Apoptosis Induction in Skin Melanoma and Lung Adenocarcinoma Cells

**DOI:** 10.3389/fbioe.2021.779393

**Published:** 2021-12-09

**Authors:** Neha Kaushik, Hyunji Oh, Yeasol Lim, Nagendra Kumar Kaushik, Linh Nhat Nguyen, Eun Ha Choi, June Hyun Kim

**Affiliations:** ^1^ Department of Biotechnology, College of Engineering, The University of Suwon, Hwaseong, South Korea; ^2^ Department of Electrical and Biological Physics, Plasma Bioscience Research Center, Kwangwoon University, Seoul, South Korea; ^3^ Laboratory of Plasma Technology, Institute of Materials Science, Vietnam Academy of Science and Technology, Hanoi, Vietnam

**Keywords:** growth inhibition, *hibiscus syriacus*, *cinnamomum loureirii* nees, lung adenocarcinoma, nanoemulsions, skin melanoma cell line

## Abstract

Carcinogenesis is a major concern that severely affects the human population. Owing to persistent demand for novel therapies to treat and prohibit this lethal disease, research interest among scientists is drawing its huge focus toward natural products, as they have minimum toxicity comparable with existing treatment methods. The plants produce secondary metabolites, which are known to have the anticancer potential for clinical drug development. Furthermore, the use of nanocarriers could boost the solubility and stability of phytocompounds to obtain site-targeting delivery. The identification of potential phytochemicals in natural compounds would be beneficial for the synthesis of biocompatible nanoemulsions. The present study aimed to investigate the potential cytotoxicity of ethanol extracts of *Hibiscus syriacus* and *Cinnamomum loureirii* Nees plant parts on human skin melanoma (G361) and lung adenocarcinoma (A549) cells. Importantly, biochemical analysis results showed the presence of high phenol (50–55 µgGAE/mg) and flavonoids [42–45 µg quercetin equivalents (QE)/mg] contents with good antioxidant activity (40–58%) in *C. loureirii* Nees plants extracts. This plant possesses potent antiproliferative activity (60–90%) on the malignant G361 and A549 and cell lines correlated with the production of nitric oxide. Especially, *C. loureirii* plant extracts have major metabolites that exhibit cancer cell death associated with cell cycle arrest. These findings support the potential application of *Cinnamomum* for the development of therapeutic nanoemulsion in future cancer therapy.

## Introduction

Despite advancements in cancer research, diagnosis, and therapy, cancer disease remains a serious health issue and a leading cause of death worldwide. Up to now, many studies of the potential health benefits of phytoconstituents and their feasible biomedical applications as food and pharmaceutical supplements have been discovered (Mishra et al., 2018; Sharifi-Rad et al., 2018). A comprehensive preclinical oncology study has indicated that plant-derived molecules could be administered as both isolated component and whole foods that undoubtedly affects carcinogenesis, including breast cancer (Kubatka et al., 2016; Kapinova et al., 2017; Kubatka et al., 2017). Phytochemicals have been proven effective as a strong antioxidant, immunomodulator, and anti-inflammatory agent detected by *in vitro* and *in vivo* experiments. In tumor-related studies, preclinical reports verified that several plant-derived substances (separated or as combinations) are capable of blocking carcinogenesis processes through inhibition of multiple targets in the tumor microenvironment, eventually suppressing anticancer molecular signaling pathways without undesirable adverse effects that usually occur with the traditional chemotherapies (Ham et al., 2015; Pistollato et al., 2015; Battino et al., 2019). Furthermore, numerous plant-derived molecules have been evidenced to control the angiogenesis, cell cycle, apoptosis, and activity of cancer stem cells in cancer tissue for malignant growth suppression in organisms (Kapinova et al., 2018; Kapinova et al., 2019). In contrast, it has been seen that the use of these plant products can improve therapeutic and radiotherapy efficiency and also reduce the toxicity of manufactured drugs when they were given concurrently (Chang et al., 2015; Szejk et al., 2016). Additionally, studies have been shown that nanotechnology-based drug delivery systems applied for delivering natural compounds have substantial advantages for cancer therapy (Navya et al., 2019). These reports suggest that nanoparticles can increase the uptake of water-insoluble phytochemicals and improve the route of these natural agents across cell membranes (Duan and Li, 2013), thereby improving selective cancer cell killing by sustained drug release (Rawal and Patel, 2019).

*Cinnamomum loureirii* Nees (known as Vietnamese cinnamon) is a persistent plant of the family Lauraceae. *Cinnamomum* extracts, regardless of the species, have been accompanying a wide variety of health benefits. In ancient times, several edible plants were consumed as medicinal therapies in many countries due to the presence of various secondary metabolites, frequently called phytochemicals. Especially, *C. loureirii* is widely used as medicine in Korea. The inner bark of *C. loureirii* is acquired from the trees commonly used as a flavoring and spice agent (Li et al., 2010). It contains large quantities of bioactive components, such as tannins, essential oils, and coumarins. Many studies suggested that *Cinnamomum* has potent antioxidant activity (Mathew and Abraham, 2006) and antimicrobial activity (Ooi et al., 2012) and also plays a significant role in controlling lipid and glucose levels (Anderson, 2008; Anderson, 1997). Additionally, *C. loureirii* has been discovered to be applicable for the cure of inflammatory diseases, dyspepsia, gastritis, and blood circulation disorders and could retain analgesic, antipyretic, anti-ulcerogenic, and anti-allergic effects (Kurokawa et al., 1998; Zhu et al., 1993; Yuan et al., 2017). Interestingly, *Cinnamomum*-synthesized gold nanoparticles can serve as outstanding computed tomography/photoacoustic agents for tumor recognition through nano-pharmaceuticals (Chanda et al., 2010). To the best of our knowledge, no study has discovered the major components in *C. loureirii* Nees and its inhibitory effects on anticancer activity utilizing *in vitro* cell line models using different cell types. Another study showed that *Cinnamomum verum* essential oil had been proven against *Trypanosoma cruzi* that eventually interferes with the parasite differentiation process *in vitro* (Azeredo et al., 2014). Also, Wen et al. (2018) mentioned the use of *Cinnamomum osmophloeum* Kanehira leaf extracts for the treatment of hair loss and growth due to the presence of cinnamic acid and cinnamic aldehyde Wen et al. (2018).

*Hibiscus syriacus* belongs to the family Malvaceae that is known for its anthelmintic, antipyretic, and antifungal characteristics (Maganha et al., 2010). Previously, *H. syriacus* extracts have been shown to induce antioxidant activity (Kwon et al., 2003). Interestingly, all parts of *H. syriacus,* including fruit, stem, root, flower, and skin, show good therapeutics effect, thereby widely used as herbal medicinal in Asian countries. In 2008, Cheng et al. (2012) revealed that extracts prepared from *H. syriacus* skin could activate p53 for lung cancer cell apoptosis through activation of the apoptosis-inducing factor pathway. Besides, more recently, *H. syriacus*-synthesized gold nanoparticles were shown to act as a probable autophagy inducer for lipopolysaccharide-triggered macrophages inflammation, offering an innovative perception in the management of inflammation-related disorders (Xu et al., 2021).

This study aims to identify the activity of *H. syriacus* and *C. loureirii* for induction of cancer cell apoptosis. To investigate the cytotoxic effect of these plants, the ethanolic extracts were prepared and subjected to the treatment of cancer cells. To isolate the potential list of compounds from the selected candidate, liquid chromatography quadrupole time-of-flight mass spectrometry (LC-QTOF-MS) analysis was used in this study. The data obtained indicated that approximately 6,500 compounds were tentatively dispersed in the *C. loureirii* stem extracts, which have the strong presence of 11 major phytochemicals. Our data indicated the potential use of *C. loureirii* stem extracts as a natural anticancer product and offer evidence for the presence of vanilloloside and epicatechin as major potent agents for their use in health problems associated with cancer.

## Materials and Methods

### Plant Materials

The plants used in this study are grown at the garden of Woori flowers at Gwacheon City, Gyeonggi-Do, Republic of Korea (Latitude-37.335224°N, longitude 126.822052°E). Experimental research on plants complies with relevant institutional, national, and international guidelines and legislation. The stem of the *Hibiscus* plants used in the study was collected under the permission of the Research Institute of Woori Flowers. At the same time, *Cinnamomum* plants were collected under permission from the Korea Tropical Plant Research Center, Cheju Island, Republic of Korea. These plants were identical to a specimen deposited to the National Wild Plant bank, National Institute of Biological Resources, Ministry of Environment, Republic of Korea. These plant families and parts used are briefly outlined in Table 1.

**TABLE 1:** Overview of plant extracts used in this study.

Plant	Family	Location	Part used
*Hibiscus syriacus* “Columbine”	Malvaceae	Korea	Twig
*Hibiscus syriacus*	Malvaceae	Korea	Twig
“Hanol-tanshim”			
*Hibiscus syriacus*	Malvaceae	Korea	Twig
Champion			
*Hibiscus syriacus*	Malvaceae	Korea	Twig
Taehwagang			
*Cinnamomum loureirii* Nees	Lauraceae	Korea	Twig, Leaves

### Extract Preparation

**FIGURE 1:**
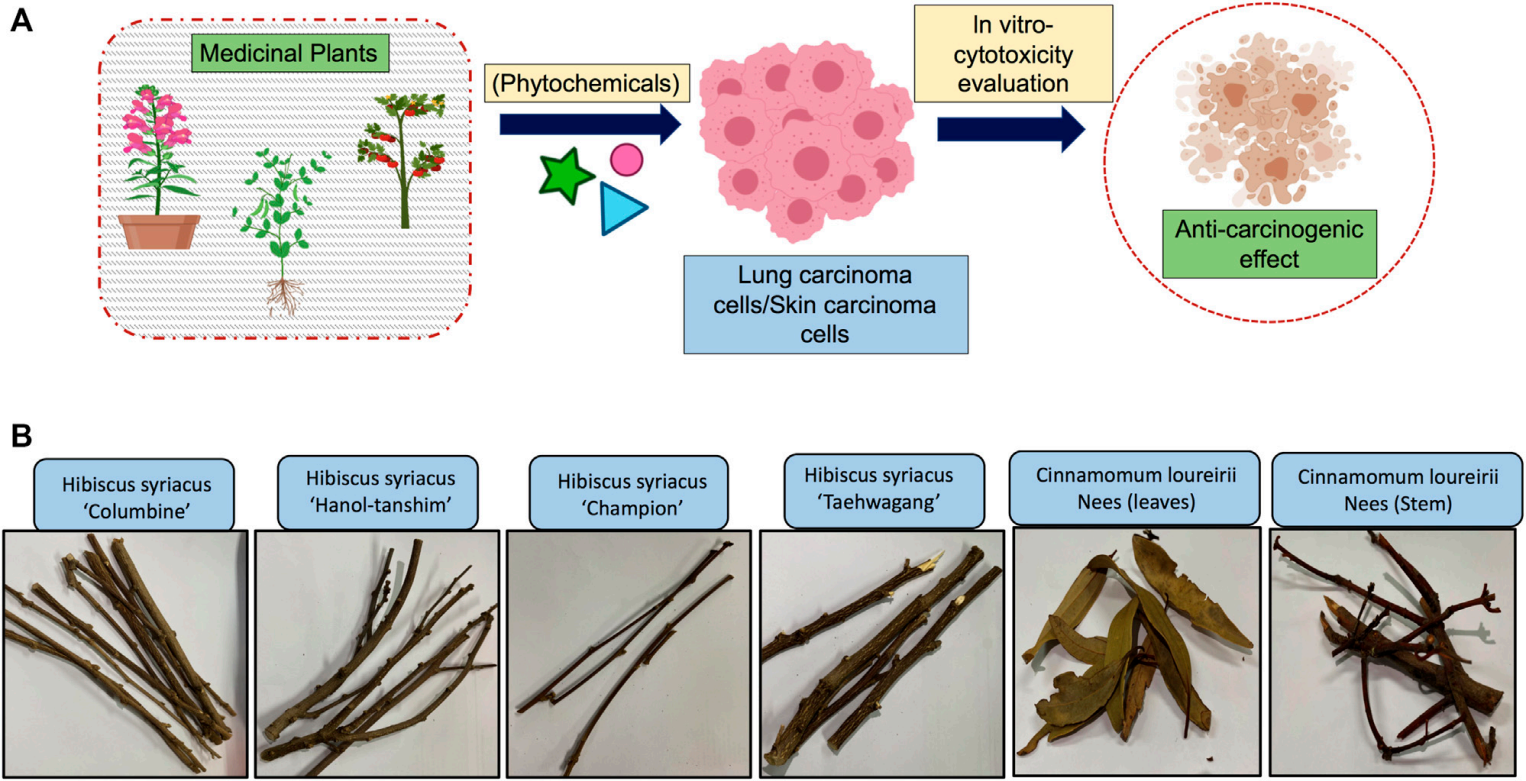
Proposed experimental plan and material used in this study. **(A)** Schematic representation of proposed study plan. **(B)** Pictures of plant material (*Hibiscus syriacus* and *Cinnamomum loureirii* Nees) used for preparation of extracts.

For extract preparation, each plant portion was collected and powdered with a blender and then placed in 70% EtOH in a shaking incubator for the next 2 days. Next, all the procedures were performed similarly as described in our earlier report (Kaushik et al., 2020). Cell experiments were performed using various obtained extracts dilutions to analyze anticancer properties (Figure 1).

### Cell Culture and Extract Treatments

Human lung carcinoma A549 and skin melanoma G361 cells were bought from the Korean Cell Line Bank (Korea). Both the A549 and G361 cancer cells were cultured in Dulbecco's modified Eagle medium and Roswell Park Memorial Institute-1640 media, supplemented with 10% fetal bovine serum, 100 μg/ml of streptomycin, and 100 U/mL of penicillin (Gibco, Waltham, MA, United States). These cells were kept in a humidified incubator at 37°C containing 5% CO_2_ and regularly passaged each 2–3 days. Stock concentrations were prepared in DI water for desired cell experiments.

### Radical Scavenging Activity

The plant extracts effect on DPPH (Sarikurkcu et al., 2016) radicals were assessed in *Hibiscus* and *Cinnamomum* species (Kim J. H. et al., 2016). Briefly, 1 ml of sample solution was added to 4 ml of a 0.004% DPPH prepared in methanol. Ascorbic acid (Sigma-Aldrich) was included as the standard to determine DPPH activity in the test samples. The developed color in the test sample was read at 517 nm using absorbance at room temperature after 30 min of incubation using an enzyme-linked immunosorbent assay reader (Epoch; BioTek Instruments, United States).

### Determination of Total Bioactive Components

Total phenolic and flavonoid constituents were detected using the methods as previously described (Zengin et al., 2014). To measure the phenolic contents, gallic acid (0.04–200 μg/ml) was utilized as the standard in the experiment. The concentrates of phenolic constituents that exist in *H. syriacus* and *C. loureirii* Nees were shown in milligrams of gallic acid equivalents per gram of individual extract. Nevertheless, quercetin (00.4–200 μg/ml) was used as the standard for flavonoids, and the entire flavonoid contents were shown in milligrams of QE per gram of individual extract.

### Cell Viability Assay

To detect cancer cell viability, cells were exposed with various extract concentrations (1,000, 500, 250, 125, and 62.5 μg/ml) and incubated for 48 h. After desired time point, viability was measured using alamarBlue (AB; Thermo Fisher Scientific, United States) dye. Concisely, an AB solution was added to every well, incubated for the next 2 h, and measured as described in our earlier work (Nguyen et al., 2021). Observed fluorescence was reflected as a measure of AB dye conversion in the untreated and treated cell samples.

### Cell Cycle Arrest

Briefly, 48 h of post-extract treatments, the cells were collected and rinsed with ice-cold phosphate-buffered saline continued by fixation with 70% cold EtOH at 4°C for 10–12 h. Subsequently, the cells were washed further, resuspended in a cell cycle staining solution including 1 μg/ml RNase A with 5 μg/ml propidium iodide (PI), and incubated for 25–30 min in the dark place (Bhartiya et al., 2021). Immediately, the stained cells were examined using a flow cytometer (BD FACSVerse; United States) with FACSuite software. In every test sample, 10,000 events/samples were identified.

### Nitric Oxide Measurement (Griess Assay)

The amount of nitric oxide (NO) formed was calculated from the aggregation of the nitrite (NO_2_^−^, steady NO metabolite) by Griess reagent assay. In this experiment, the 100-µl culture supernatant was collected from untreated and treated samples and mixed with 100-µl Griess reagent, and the absorbance was accessed at 540 nm (Green et al., 1982). The amount of nitrite was calculated using the standard curve.

### Phytoconstituents Characterization

#### Fourier-Transform Infrared and Ultraviolet-Visible Spectroscopy Analysis

Ultraviolet-Visible (UV-Vis) absorption spectroscopy was carried out using a J-815 spectrophotometer (JASCO, Japan). Fourier transformed infrared (FTIR) spectroscopy was performed using a Shimadzu QATR-S spectrometer (Kyoto, Japan). For both UV-Vis and FTIR measurements, the *C. loureirii* Nees extracts were dissolved in methanol.

#### Liquid Chromatography Quadrupole Time-Of-Flight Mass Spectrometry Analysis

The *C. loureirii* Nees stem extracts were analyzed at the ideal conditions using LC-QTOF-MS supplied with PDA detector (Waters, United States) and asymmetry C18 column of 100 × 2.1 mm, 1.8 mm particle size (Waters, United States). The *C. loureirii* Nees stem extracts were prepared in high-performance liquid chromatography grade pure methanol to produce 20 ppm. For analysis, the mobile phases used as A (water with 0.1% formic acid) and B (100% acetonitrile). The gradients elution were used as earlier (Alara et al., 2018), using the injection volume of 20 ml and flow rate of 0.5 ml/min. The bioactive constituents in the *C. loureirii* Nees stem extracts were tentatively designated with Waters UNIFY Software 1.0.0 (Alara et al., 2018).

### Statistical Analysis

All results are expressed as the means ± standard deviation of triplicate assessments from three independent experiment sets. Significant differences between groups were examined using the Student's t-test in PRISM 9 software. Multiple group comparisons were evaluated using analysis of variance. Levels of significance are mentioned by *p < 0.05, **p < 0.01, and ***p < 0.001.

## Results

### Determination of Phytoconstituents Levels in *Hibiscus syriacus* and *Cinnamomum loureirii* Nees Plant Extracts

**FIGURE 2:**
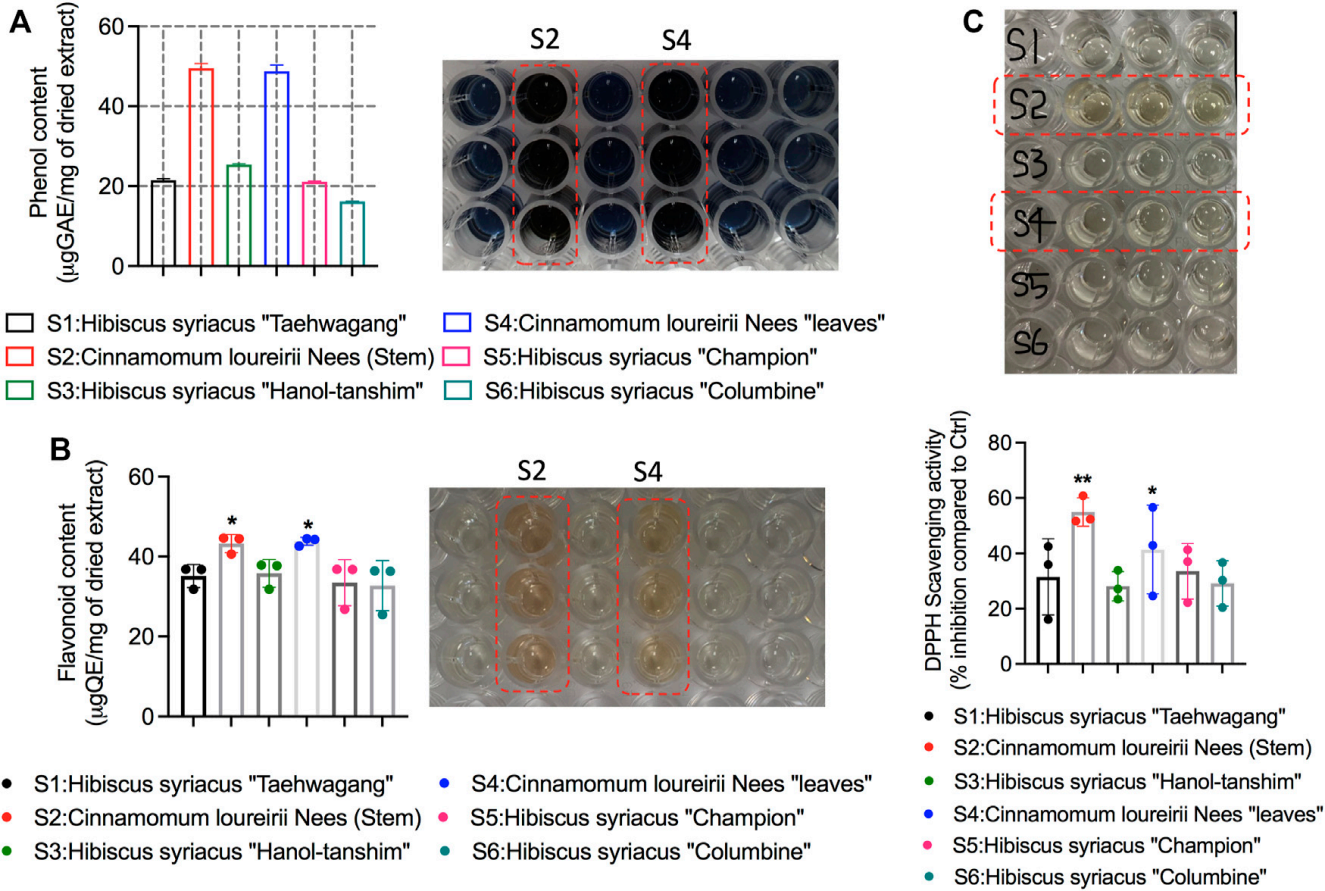
Analysis of phytochemical components and antioxidant activity in *Hibiscus* and *Cinnamomum loureirii* Nees plant extracts. **(A)** detection of total phenol contents, **(B)** Detection of flavonoid contents, and **(C)** antioxidant scavenging activity by DPPH assay. All these ethanolic extracts were used at 1 mg/ml concentration. *p < 0.05; **p < 0.01.

Before performing cell experiments, the first objective of this study was to examine all the plant ethanolic extracts for their phytoconstituent levels. Primary screening of all plant species of *H. syriacus* and *C. loureirii* Nees extracts (described in Table 1) showed the existence of phytochemical constituents at different levels. These extracts were subjected to qualitative biochemical assessments to identify secondary metabolites such as total phenols and flavonoids. Higher levels of phenols and flavonoids were detected in *Cinnamomum* plant extracts over *Hibiscus* plant species extracts, as shown in Figure 2A. Colorimetric visualization demonstrated the high intensity of color production in *Cinnamomum* extracts only (Figure 2B). It has been shown that plants possess antioxidant activity mainly because of the presence of phenolic compounds, including flavonoids and polyphenols. Flavonoids are believed to be key antioxidants in traditional herbal medicine (Dragland et al., 2003) (Pietta, 2000). Interestingly, *Cinnamomum* ethanolic extracts have shown augmented antioxidant activity (55%) as observed by DPPH free radical inhibition, which was well correlated with their increased phytoconstituents levels. Notably, *Cinnamomum* stem ethanolic extracts have higher antioxidants activity as compared with leaf ethanolic extracts (Figure 2C).

### Chemical Profiling of *Cinnamomum loureirii* Nees Stem and Leaves Using Fourier Transformed Infrared, Ultraviolet-Visible, and Liquid Chromatography Quadrupole Time-Of-Flight Mass Spectrometry Analysis

**FIGURE 3:**
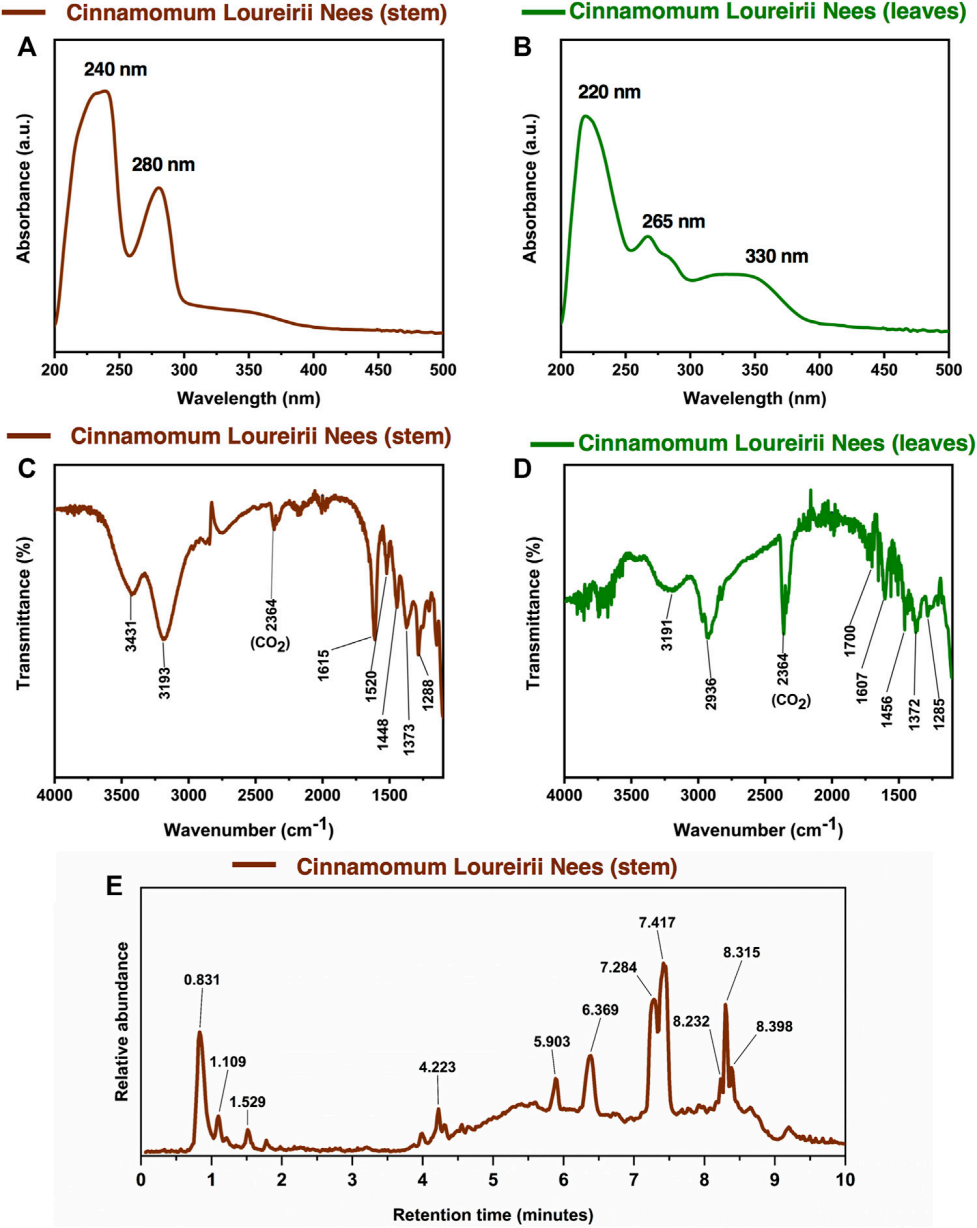
Chemical characterization of *Cinnamomum loureirii* Nees plant extracts. **(A, B)** Analysis of UV-Vis absorption spectrum of *Cinnamomum* stem and leaf extracts, respectively. **(C, D)** FTIR spectroscopy spectrum of *Cinnamomum* stem and leaf extracts, respectively. **(E)** LC-Q-TOF-MS chromatogram for tentatively assigned compounds in *Cinnamomum* stem extracts.

Plant extracts are complex mixtures of multiple phytochemicals that are difficult to identify specifically. Further to study the chemical composition of the *C. loureirii* Nees extracts, we have performed several characterizations using different chemical analysis techniques. Figure 3A shows the UV-Vis spectrum of *Cinnamomum* stem ethanolic extracts, revealing two absorption peaks located at 240 and 280 nm (Figure 3A). On the other hand, the leaf ethanolic extracts demonstrate the absorption peaks at 220 and 266 nm, together with a board band at 330 nm (Figure 3B).

Both *Cinnamomum* stem and leaf extracts possess strong absorption in the range of 200–400 nm, indicating the existence of flavonoids and their derivatives in the extracts (Jurasekova et al., 2006). Afterward, FTIR spectroscopy was carried out to identify the functional groups present in the *C. loureirii* extracts. Figure 3C shows the vibration absorption bands observed in the *Cinnamomum* stem extracts, i.e., OH stretching (3,431, 3,190 cm^−1^), C=C stretching (1,615 cm^−1^), N-O stretching (1,520 cm^−1^), C-H bending (1,448 cm^−1^), S=O stretching (1,373 cm^−1^), and C-O stretching (1,288 cm^−1^) groups. The FITR spectrum of the *Cinnamomum* stem extracts is shown in Figure 3D, with the presence of OH stretching (3,191 cm^−1^), *C-H stretching* (2,936 cm^−1^), *C=O stretching* (1700 cm^−1^), *C=C stretching* (1,615 cm^−1^), *C-H bending* (1,456 cm^−1^), *S=O stretching* (1,372 cm^−1^), and *C-O stretching* (1,285 cm^−1^) groups. Table 2 summarizes the details of functional groups in *Cinnamomum* stem and leaf extracts detected by FTIR spectroscopy. Figure 3E shows the LC-QTOF-MS analysis of the *C. loureirii* Nees stem extracts. The details of identified phytochemicals are listed in Table 2. We noted that the extract of *Cinnamomum loureirii* Nees contains an enormous number of phytochemicals; thus, it is difficult to list all the existing compounds. Herein, 11 significantly separated compounds from the chromatography were mentioned. For instance, several sugar molecules were detected, such as D-1-[(3-carboxypropyl)amino]-1-deoxyfructose (RT: 0.831 min), vanilloloside (RT: 1.529 min), and (1x, 2x)-guaiacyl glyceryl 3-glucoside (RT: 0.831 min). Flavonoids and polyphenols and are also presented in the extract, i.e. epicatechin (RT: 4.223 min), (7R, 8R,E)-8-methyl-6-(2-methylpropylidene)octahydroindolizine-7,8-diol (RT: 5.903 min) (1R,2S,4R, 8R)-p-menthane-1,2,8,9-tetrol. The MS detection of flavonoids and polyphenols agrees with the FTIR and UV-Vis results.

**TABLE 2:** FTIR functional groups present in *Cinnamomum loureirii* stem and leaf extracts.

Functional groups	Absorption (cm^−1^)- stem	Absorption (cm^−1^)- leaves
OH stretching	3,431, 3,190	3,191
C-H stretching	-	2,936
C=O stretching	-	1700
C=C stretching	1,615	1,607
N-O stretching	1,520	-
C-H bending	1,448	1,456
S=O stretching	1,373	1,372
C-O stretching	1,288	1,285

### Evaluation of Antiproliferative Activity *Cinnamomum loureirii* Nees Plant Extracts Against A549 Human Lung Adenocarcinoma and G361 Skin Melanoma Cell Lines

**FIGURE 4:**
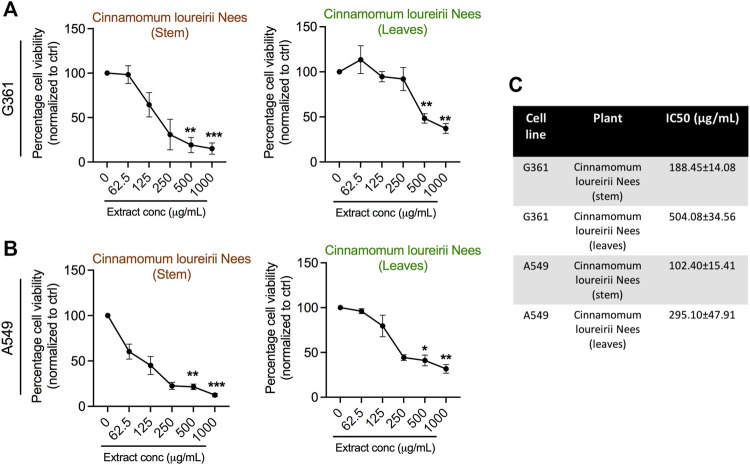
Assessment of cellular metabolic viability in human skin melanoma G361 and A549 lung cancer cells. **(A, B)** AlamarBlue assay was implemented in G361 and A549 cancer cells treated with various concentrations (0, 62.5, 125, 250, 500, and 1,000 μg/ml) of *Cinnamomum loureirii* Nees plant extracts after 48 h of incubation. **(C)** IC_50_ values of *Cinnamomum loureirii* Nees extracts in both cancer cell lines as indicated panels. These IC_50_ values were calculated using PRISM software. *p < 0.05; **p < 0.01; ***p < 0.001. No extract (0 μg/ml) treated sample was considered as controls for all tested plant extracts.

To investigate the antiproliferative potential of *Cinnamomum* plant extracts prepared from both stem and leaves, we have tested different concentrations of these extracts against G361 skin cancer and A549 lung cancer cells to check their effect on different tissue types. After 24 h of cell attachment, both the cells were exposed with 0, 62.5, 125, 250, 500, and 1,000 μg/ml concentrations of plant extracts and incubated for the further 48 h to check their effect on cell survival. The result of the alamarBlue assay revealed that both *Cinnamomum* stem and leaf extracts gradually declined the survival percent of G361 and A549 cancer cells as the concentration increased (Figures 4A,B). *Cinnamomum* stem and leaf ethanolic extracts exhibited IC_50_ values of 188.45 and 504.08 μg/ml, respectively, in G361 skin cancer cells. In the case of A549 lung cancer cells, *Cinnamomum* stem and leaf ethanolic extracts exhibited IC_50_ values of 102.40 and 295.10 μg/ml, respectively, in G361 skin cancer cells. Remarkably, *Cinnamomum* stem extracts have a higher potential to suppress cancer cell viability over leaf extracts regardless of tissue-specific cell type. Taken together, these alamarBlue results suggest that *Cinnamomum* plant extracts were able to inhibit the growth of cancer cells in a concentration-dependent fashion.

### NO Production by *Cinnamomum loureirii* Nees Plant Extracts Against Cancer Cell Lines

**FIGURE 5:**
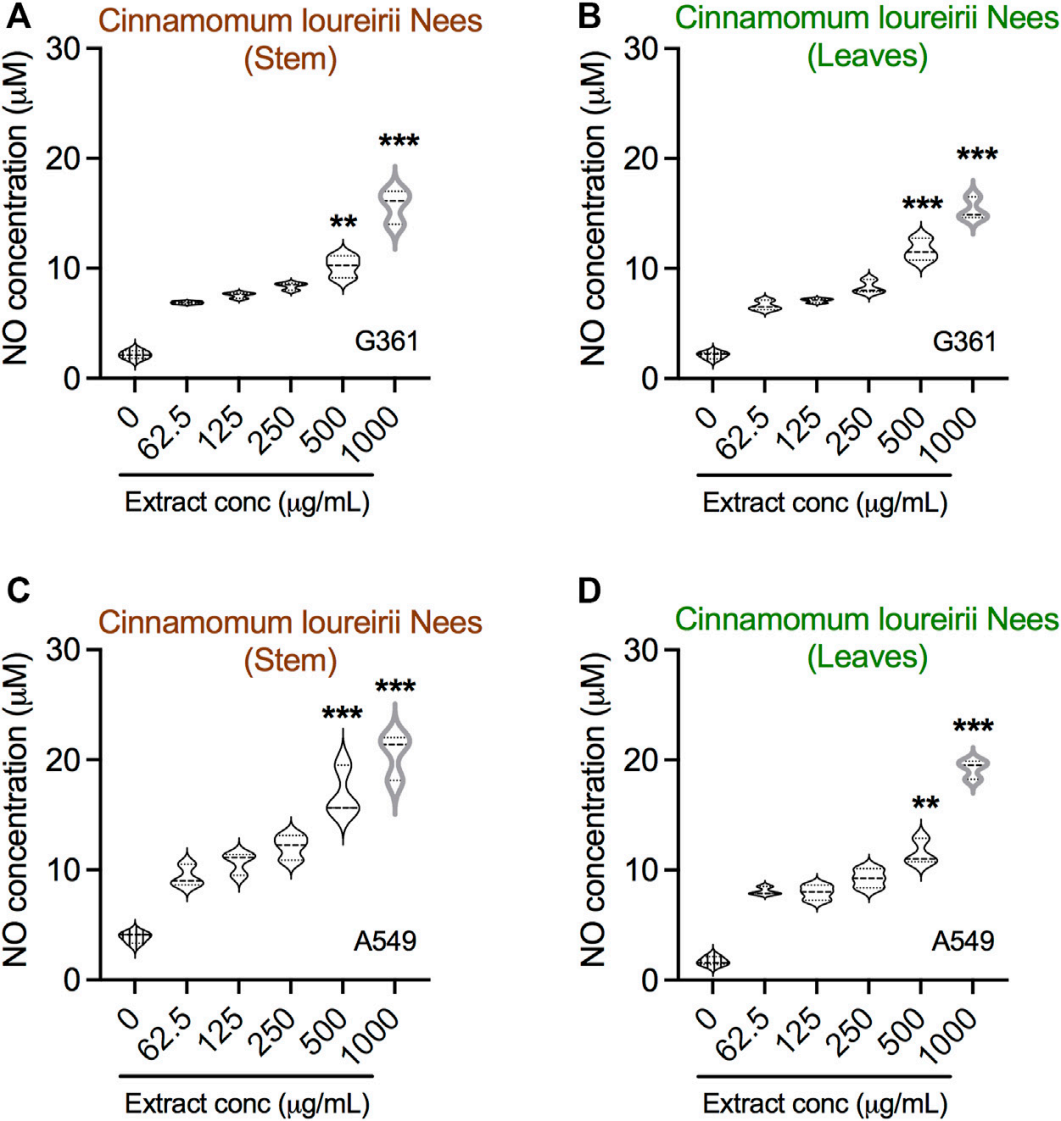
Detection of nitric oxide in *Cinnamomum loureirii* Nees plant extracts treated cancer cells. **(A–D)** Assessment of NO in G361 and A549 cancer cells treated with various concentrations (0, 62.5, 125, 250, 500, and 1,000 μg/ml) of *Cinnamomum loureirii* Nees stem and leaf extracts after 48 h, respectively. **p < 0.01; ***p < 0.001. No extract (0 μg/ml) treated sample was considered as controls for each case.

⋅NO has been shown to play important roles in cancer biology, including the innate immune response, neovascularization, cancer metastasis, and cell death (Wink et al., 1998); we next questioned where our *Cinnamomum* stem and leaf plant ethanolic extracts were able to generate NO in cancer cells. To this end, cell supernatant was collected from G361 and A549 cancer cells after treatment at various concentrations, and all samples were subjected to Griess assay for NO detection. Remarkably, with the increase in concentrations, the quantity of NO generation was significantly enhanced, as seen in both G361 and A549 cancer cells (Figures 5A–D). These effects were similar in both stem and leaf extracts when applied to G361 and A549 cancer cells.

### Apoptosis Cancer Cell Death Induction by Cell Cycle Arrest by *Cinnamomum loureirii* Nees Plant Extracts

**FIGURE 6:**
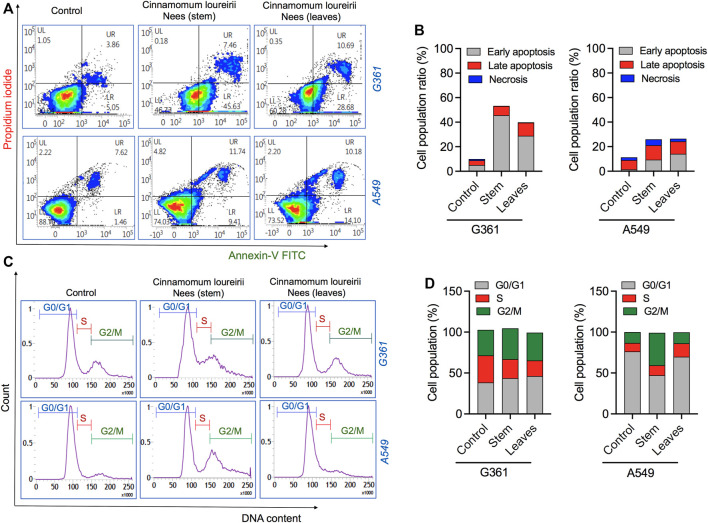
Analysis of cell death induced by *Cinnamomum loureirii* Nees plant extracts in cancer cells. **(A, B)** Flow cytometric analysis of apoptotic cell death in G361 and A549 cancer cells using Annexin-V FITC/PI treated with IC_50_ concentrations (calculated from Figure 4C) of *Cinnamomum loureirii* Nees stem and leaf extracts after 48 h, respectively. **(C, D)** Flow cytometric analysis of cell cycle arrest in G361 and A549 cancer cells using PI/RNase staining treated with IC_50_ concentrations (calculated from Figure 4C) of *Cinnamomum loureirii* Nees stem and leaf extracts after 48 h, respectively. No extract (0 μg/ml) treated sample was considered as control for each case.

Furthermore, based on the activity of *Cinnamomum* plant extracts in growth inhibition and/or cell death of cancer cells, we investigated the ability of these ethanolic extracts to induce apoptosis in cancer cells. To check the apoptosis phenomenon, both the cells were treated with stem and leaf extracts at IC_50_ concentrations, which were calculated from alamarBlue assay results (see Figure 4C). Flow cytometry results indicated that both the extracts were able to stimulate apoptotic cell death in G361 and A549 cancer cells. However, this effect was more prominent in G361 skin melanoma cells. It was noted that stem extracts promote a maximum range of 45.63 and 7.63% cell population in early and late apoptotic phases, respectively. Simultaneously, stem and leaves both can induce apoptosis in A549 cancer cells, but necrosis was also seen (Figures 6A,B). Next, to study whether the inhibitory effects of both stem and leaf extracts were associated with cell cycle alteration, we executed cell cycle analysis in G361 and A549 cancer cells using FACS analysis after 48 h after treatment at IC_50_ concentrations of both extracts. Our G361 cell data showed that stem and leaf extract-induced cytotoxicity was related to the expansion of cell population in the G0/G1 phase and a significant decrease in S phases. In contrast, A549 cells treated with stem extracts displayed a noticeable accumulation of cells in the G2/M phase along with a reduction in the G0/G1 phase. However, this effect was not comparable in the case of A549 cells treated by leaf extract (Figures 6C, D). These results suggest that both *Cinnamomum* stem and leaf extracts affect differentially on cell tissue type regarding cell cycle arrest.

## Discussion

Earlier, herbal medicine has gained attraction for cancer treatment due to their phytochemical contents having numerous biological activities (Mann, 2002). Currently, natural products or their structural derivatives contain approximately 45–55% of the drugs used for cancer chemotherapies. Using the human genome project, researchers could identify selective gene targets for innovative anticancer drugs and pharmaceutical business challenges to attain these drug formulations *via* the use of chemistry and high-performance screening methods. Nonetheless, the large libraries of compounds fail to provide the essential formation needed for the formulation of new anticancer drugs. To overcome these problems, the much more elusive use of natural-product patterns shared with chemistry to fabricate selective analogs could have a great rate of success. Several pieces of literature provide information on the crucial role of natural products in the development of the novel anticancer candidates, and their significance in the optimization of unique molecular leads from natural products (Cragg and Newman, 2009; (Demain and Vaishnav, 2011; Basmadjian et al., 2014). The phytogenic nanoparticles are widely considered as antimicrobial agents, wound healing processes in cancer therapy, drug delivery approaches, and bioenergy or biosensors applications (Augustine and Hasan, 2020; Verma and Rani, 2021). A recent report suggests that zinc oxide nanoparticles synthesized using *Deverra tortuosa* plant extracts have the potential to induce a cytotoxic effect in different tissue-specific cancer cells (Selim et al., 2020). Similarly, the formation of silver nanoparticles using *Sesbania grandiflora* leaf extracts has been shown to be cytotoxic against breast carcinoma cells (Das et al., 2013). Magnetic iron oxide nanoparticles synthesized from *Phyllanthus niruri* were also found to be biocompatible and have high antibacterial efficacy against both Gram-positive and Gram-negative bacterial strains (Sheel et al., 2020). A similar group of researchers also highlights the importance of green synthesized nanoparticles prepared by leaf extract of *Andrographis peniculata* against zebrafish embryos as shown by *in vivo* analysis (Kumari et al., 2019) and the use of hybrid silver nanoparticles synthesized using leaf extracts of *Calotropis gigantea* in cancer cells death (Verma et al., 2016).

**FIGURE 7:**
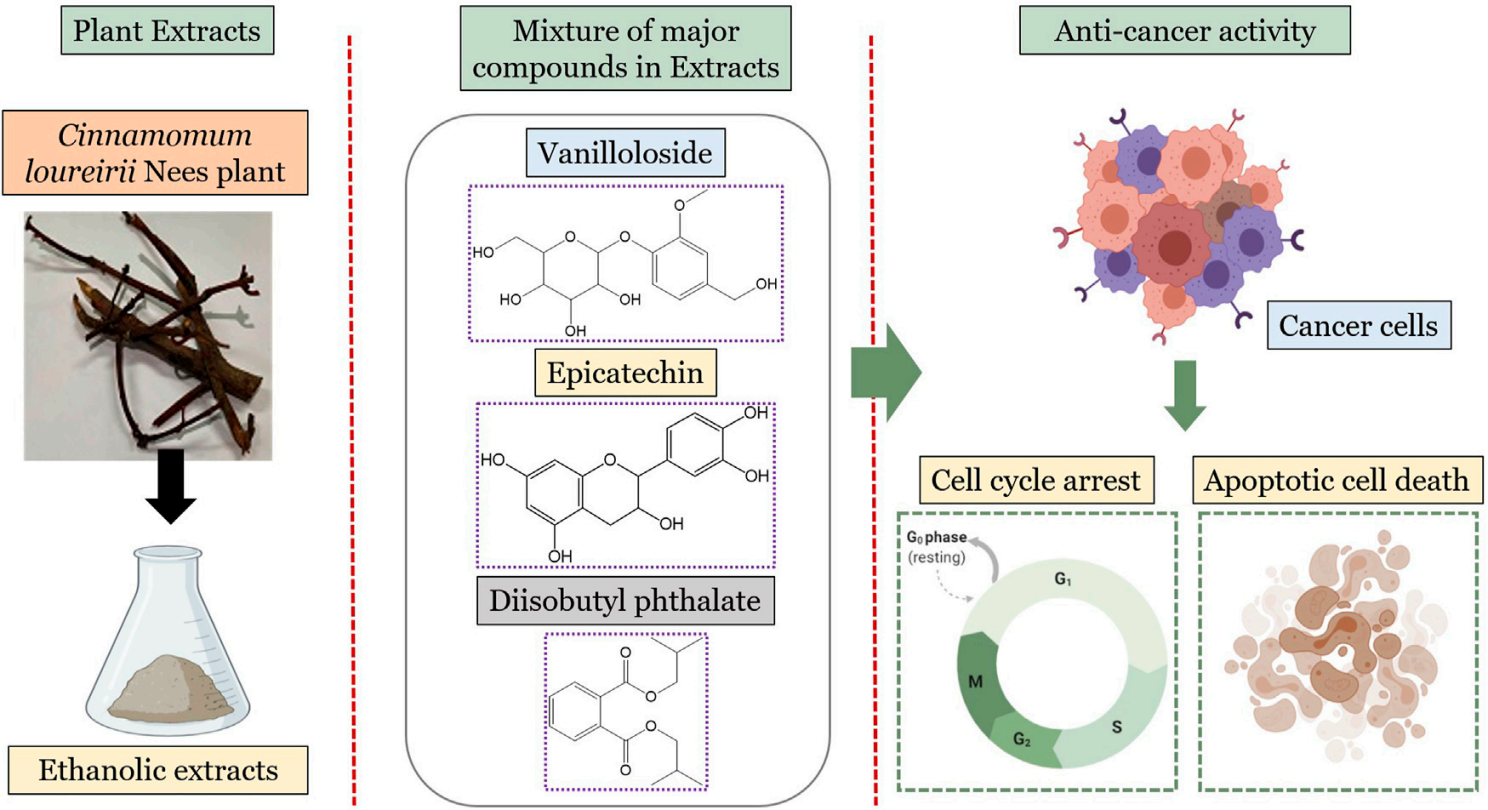
Overview of major metabolites present in *Cinnamomum loureirii* Nees plant stem extracts and their effect on anti-cancer activity.

Cinnamon is a form of spice that has been largely utilized from ancient times in most countries (Dutta and Chakraborty, 2018). The function of this spice has been broadly studied in its benefits for human health. Generally, plant products contain polyphenols (Chang, 2002); accordingly, we have seen that our *C. loureirii* Nees plant stem and leaf extracts have a mixture of numerous phytochemicals as determined by chemical analysis techniques, including FTIR and UV-Vis spectroscopy. Moreover, FTIR analysis shows the potential functional groups of the major compounds existing in plant extracts (Figures 3A–D). Table 2 provides more clarity on the functional groups present in the stem and leaf ethanolic extracts of *C. loureirii* Nees plant. It has been stated that *Cinnamomum* has anticancer properties *via* many molecular signaling mechanisms (Kwon et al., 2019). The main ability of any potential anticancer drug is its efficiency in inhibiting the growth of cancer cells. Schoene et al. (2005) showed that *Cinnamomum* extracts could promote antiproliferative effects in different blood cancer cell types. Besides, the essential oil of *Cinnamomum* has been revealed to decrease receptor tyrosine kinase activities in squamous cell carcinoma, reducing the tumor burden in those cells (Yang et al., 2015). In our study, we observed that our *C. loureirii* Nees plant stem and leaf extracts were able to suppress cancer cell death in skin melanoma and lung carcinoma cells (Figure 4). It is worth mentioning that it is the first study on cancer cell inhibition using *C. loureirii* Nees plant ethanolic extracts to the best of my knowledge. In another study, *C. loureirii* extracts are beneficial as a neuroprotective agent by preventing Alzheimer's disease. They identified that potent acetylcholine inhibitors were present in their *C. loureirii* ethanolic extracts (Kim C. R. et al., 2016). Earlier, it has been reported that phenolic components often exist in wild plants (Rana and Blazquez, 2007). Using liquid chromatography–mass spectrometry analysis, we observed that our *C. loureirii* Nees ethanolic stem extracts have a major amount of compounds (Figure 7) such as vanilloloside [as a wound-healing (Harikarnpakdee and Chowjarean, 2018), neuroprotector (Jung et al., 2010)], epicatechin [as an anticancer agent (Takanashi et al., 2017), antioxidant (Caro et al., 2019), antidiabetic (Kim et al., 2004)], diisobutyl phthalate [as a water-based adhesive, estrogenic (Harris et al., 1997) DNA damage inducer (Sicińska et al., 2021)], and many more described in Table 3.

**TABLE 3:** Phytochemicals identified in *Cinnamomum loureirii* Nees stem by LC-QTOF-MS analysis.

RT (min)	Compound	Formula	Chemical structure	Species	m/z	Error (ppm)
0.831	D-1-[(3-Carboxypropyl)amino]-1-deoxyfructose	C_10_H_19_NO_7_	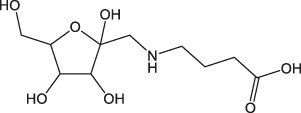	(M + H)+	226.12	1.9
1.109	(1x,2x)-Guaiacyl glyceryl 3-glucoside	C_16_H_24_O_10_	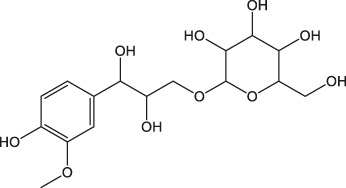	(M + Na)+	339.13	0.06
1.529	Vanilloloside	C_14_H_20_O_8_	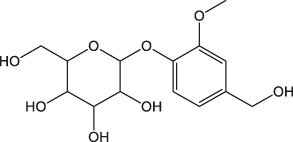	(M + Na)+	339.1	1.11
4.223	Epicatechin	C_15_H_14_O_6_	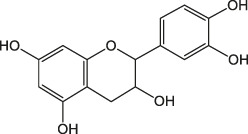	(M + H)+	291.08	1.55
5.903	(7R,8R,E)-8-Methyl-6-(2-methylpropylidene)octahydroindolizine-7,8-diol	C_13_H_23_NO_2_	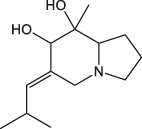	(M + H)+	226.18	1.92
6.369	(1R,2S,4R,8R)-p-Menthane-1,2,8,9-tetrol	C_10_H_20_O_4_	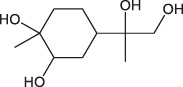	(M + Na)+	227.12	3.83
7.284	xi-5-Hydroxydodecanoic acid	C_12_H_24_O_3_	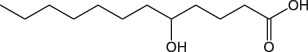	(M + H)+	217.18	2.5
7.417	5-Methylheptan-3-one	C_8_H_16_O	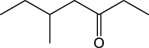	(M + H)+	129.12	3.27
8.232	PS(P-16:0/0:0)	C_22_H_44_NO_8_P	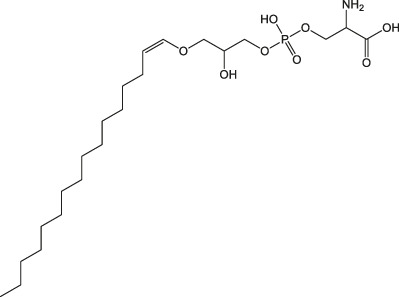	(M + NH_4_)+	499.31	0.3
8.315	Methyl (3b,11x)-3-Hydroxy-8-oxo-6-eremophilen-12-oate	C_16_H_24_O_4_	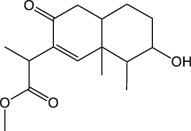	(M + H)+	281.17	1.83
8.398	Diisobutyl phthalate	C_16_H_22_O_4_	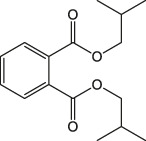	(M + Na)+(M + H)+	301.14	1.96

Recent literature indicates that NO has a potential role in cell growth inhibition and apoptosis at higher concentrations (Reveneau et al., 1999). Their study showed the antiproliferative effect in breast cancer cells could be achieved with high NO generation induced by plant extracts in a concentration-dependent manner. Similarly, our *C. loureirii* Nees plant extracts were able to increase the production of NO in both cancer cells in a dose-dependent fashion (Figure 5). This might be largely possible due to the increasing effect of the inducible nitric oxide synthase gene, which may result in the enhanced NO in cells (Alalami and Martin, 1998), thereby responsible for cell death and apoptosis through cellular damage, including DNA, protein, and other cellular components. This was consistent with our result of observed apoptotic cell death in G361 and A549 cancer cells after treatment with *C. loureirii* Nees stem and leaf ethanolic extracts. Cell cycle results showed that the effect of stem and leaf extracts was different regarding cancer cell types on cell growth arrest, suggesting them more specific for each cell cycle that has been used for treatment (Figure 6). Furthermore, it would be motivating to know the basic mechanisms involved in such cell cycle effects induced by *C. loureirii* Nees stem and leaf extracts using different cancer cell tissue types.

## Conclusion

In conclusion, the presented study showed that *C. loureirii* Nees ethanolic stem and leaf ethanolic extracts have an anticancer effect, which could be capable of inducing cell death phenomena *via* apoptosis. Moreover, our phytoconstituent characterization analysis showed the presence of polyphenols and flavonoids present in the *Cinnamomum* Nees extracts. Prominently, some vital compounds such as vanilloloside, epicatechin, and many more that exist in *Cinnamomum* extracts make them a potential candidate for health benefits. Further works are necessary to isolate particular active phytoconstituents from the extracts to be used as an efficient anticancer agent to elucidate their significance in future chemotherapies. In this regard, the application of phytochemicals in the form of nanoemulsion or nanoparticles amplifies the therapeutic effect and provides a new way to solve the difficulty in the treatment of dreadful diseases such as cancer. Therefore, the utilization of phytochemicals in nanotechnology will be a promising approach.

## Data Availability

The original contributions presented in the study are included in the article/Supplementary Material, further inquiries can be directed to the corresponding authors.

## References

[B1] Alalami O. Martin J. H. J. (1998). ZR-75-1 Human Breast Cancer Cells: Expression of Inducible Nitric Oxide Synthase and Effect of Tamoxifen and Phorbol Ester on Nitric Oxide Production. Cancer Lett. 123, 99–105. 10.1016/s0304-3835(97)00404-7 9461025

[B2] Alara O. R. Abdurahman N. H. Ukaegbu C. I. Azhari N. H. Kabbashi N. A. (2018). Metabolic Profiling of Flavonoids, Saponins, Alkaloids, and Terpenoids in the Extract From Vernonia Cinerea Leaf Using LC-Q-TOF-MS. J. Liquid Chromatogr. Relat. Tech. 41, 722–731. 10.1080/10826076.2018.1511995

[B3] Anderson R. A. (1997). Nutritional Factors Influencing the Glucose/Insulin System: Chromium. J. Am. Coll. Nutr. 16, 404–410. 10.1080/07315724.1997.10718705 9322187

[B4] Anderson R. A. (2008). Chromium and Polyphenols From Cinnamon Improve Insulin Sensitivity. Proc. Nutr. Soc. 67, 48–53. 10.1017/s0029665108006010 18234131

[B5] Augustine R. Hasan A. (2020). Emerging Applications of Biocompatible Phytosynthesized Metal/Metal Oxide Nanoparticles in Healthcare. J. Drug Deliv. Sci. Technology. 56, 101516. 10.1016/j.jddst.2020.101516

[B6] Azeredo C. M. O. Santos T. G. Maia B. H. L. d. N. S. Soares M. J. (2014). *In Vitro* Biological Evaluation of Eight Different Essential Oils Against Trypanosoma Cruzi, With Emphasis on *Cinnamomum verum* Essential Oil. BMC Complement. Altern. Med. 14, 309. 10.1186/1472-6882-14-309 25148924 PMC4155112

[B7] Basmadjian C. Zhao Q. Bentouhami E. Djehal A. Nebigil C. G. Johnson R. A. (2014). Cancer Wars: Natural Products Strike Back. Front. Chem. 2, 20. 10.3389/fchem.2014.00020 24822174 PMC4013484

[B8] Battino M. Forbes-Hernández T. Y. Gasparrini M. Afrin S. Cianciosi D. Zhang J. (2019). Relevance of Functional Foods in the Mediterranean Diet: the Role of Olive Oil, Berries and Honey in the Prevention of Cancer and Cardiovascular Diseases. Crit. Rev. Food Sci. Nutr. 59, 893–920. 10.1080/10408398.2018.1526165 30421983

[B9] Bhartiya P. Mumtaz S. Lim J. S. Kaushik N. Lamichhane P. Nguyen L. N. (2021). Pulsed 3.5 GHz High Power Microwaves Irradiation on Physiological Solution and Their Biological Evaluation on Human Cell Lines. Sci. Rep. 11, 8475. 10.1038/s41598-021-88078-x 33875781 PMC8055702

[B10] Caro A. A. Davis A. Fobare S. Horan N. Ryan C. Schwab C. (2019). Antioxidant and Pro-Oxidant Mechanisms of (+) Catechin in Microsomal CYP2E1-Dependent Oxidative Stress. Toxicol. Vitro. 54, 1–9. 10.1016/j.tiv.2018.09.001 PMC6281780 30195042

[B11] Chanda N. Shukla R. Zambre A. Mekapothula S. Kulkarni R. R. Katti K. (2010). An Effective Strategy for the Synthesis of Biocompatible Gold Nanoparticles Using Cinnamon Phytochemicals for Phantom CT Imaging and Photoacoustic Detection of Cancerous Cells. Pharm. Res. 28, 279–291. 10.1007/s11095-010-0276-6 20872051

[B12] Chang H.-P. Sheen L.-Y. Lei Y.-P. (2015). The Protective Role of Carotenoids and Polyphenols in Patients With Head and Neck Cancer. J. Chin. Med. Assoc. 78, 89–95. 10.1016/j.jcma.2014.08.010 25306067

[B13] Chang R. (2002). Bioactive Polysaccharides From Traditional Chinese Medicine Herbs as Anticancer Adjuvants. J. Altern. Complement. Med. 8, 559–565. 10.1089/107555302320825066 12470436

[B14] Cheng Y.-L. Lee S.-C. Harn H.-J. Huang H.-C. Chang W.-L. (2012). The Extract of Hibiscus syriacusInducing Apoptosis by Activating P53 and AIF in Human Lung Cancer Cells. Am. J. Chin. Med. 36, 171–184. 10.1142/s0192415x08005680 18306460

[B15] Cragg G. M. Newman D. J. (2009). Nature: a Vital Source of Leads for Anticancer Drug Development. Phytochem. Rev. 8, 313–331. 10.1007/s11101-009-9123-y

[B16] Das J. Paul Das M. Velusamy P. (2013). Sesbania Grandiflora Leaf Extract Mediated Green Synthesis of Antibacterial Silver Nanoparticles Against Selected Human Pathogens. Spectrochimica Acta A: Mol. Biomol. Spectrosc. 104, 265–270. 10.1016/j.saa.2012.11.075 23270884

[B17] Demain A. L. Vaishnav P. (2011). Natural Products for Cancer Chemotherapy. Microb. Biotechnol. 4, 687–699. 10.1111/j.1751-7915.2010.00221.x 21375717 PMC3815406

[B18] Dragland S. Senoo H. Wake K. Holte K. Blomhoff R. (2003). Several Culinary and Medicinal Herbs Are Important Sources of Dietary Antioxidants. J. Nutr. 133, 1286–1290. 10.1093/jn/133.5.1286 12730411

[B19] Duan X. Li Y. (2013). Physicochemical Characteristics of Nanoparticles Affect Circulation, Biodistribution, Cellular Internalization, and Trafficking. Small. 9, 1521–1532. 10.1002/smll.201201390 23019091

[B20] Dutta A. Chakraborty A. (2018). Cinnamon in Anticancer Armamentarium: A Molecular Approach. J. Toxicol. 2018, 1–8. 10.1155/2018/8978731 PMC5896244 29796019

[B21] Green L. C. Wagner D. A. Glogowski J. Skipper P. L. Wishnok J. S. Tannenbaum S. R. (1982). Analysis of Nitrate, Nitrite, and [15N]Nitrate in Biological Fluids. Anal. Biochem. 126, 131–138. 10.1016/0003-2697(82)90118-x 7181105

[B22] Ham S. L. Nasrollahi S. Shah K. N. Soltisz A. Paruchuri S. Yun Y. H. (2015). Phytochemicals Potently Inhibit Migration of Metastatic Breast Cancer Cells. Integr. Biol. 7, 792–800. 10.1039/c5ib00121h PMC5474751 26120051

[B23] Harikarnpakdee S. Chowjarean V. (2018). Grammatophyllum Speciosum Ethanolic Extract Promotes Wound Healing in Human Primary Fibroblast Cells. Int. J. Cell Biol. 2018, 1–6. 10.1155/2018/7836869 PMC6215563 30420887

[B24] Harris C. A. Henttu P. Parker M. G. Sumpter J. P. (1997). The Estrogenic Activity of Phthalate Esters *In Vitro* . Environ. Health Perspect. 105, 802–811. 10.1289/ehp.97105802 9347895 PMC1470189

[B25] Jung H. A. Jung Y. J. Hyun S. K. Min B.-S. Kim D.-W. Jung J. H. (2010). Selective Cholinesterase Inhibitory Activities of a New Monoterpene Diglycoside and Other Constituents From *Nelumbo nucifera* Stamens. Biol. Pharm. Bull. 33, 267–272. 10.1248/bpb.33.267 20118551

[B26] Jurasekova Z. Garcia-Ramos J. V. Domingo C. Sanchez-Cortes S. (2006). Surface-Enhanced Raman Scattering of Flavonoids. J. Raman Spectrosc. 37, 1239–1241. 10.1002/jrs.1634

[B27] Kapinova A. Kubatka P. Golubnitschaja O. Kello M. Zubor P. Solar P. (2018). Dietary Phytochemicals in Breast Cancer Research: Anticancer Effects and Potential Utility for Effective Chemoprevention. Environ. Health Prev. Med. 23, 36. 10.1186/s12199-018-0724-1 30092754 PMC6085646

[B28] Kapinova A. Kubatka P. Liskova A. Baranenko D. Kruzliak P. Matta M. (2019). Controlling Metastatic Cancer: the Role of Phytochemicals in Cell Signaling. J. Cancer Res. Clin. Oncol. 145, 1087–1109. 10.1007/s00432-019-02892-5 30903319 PMC11810423

[B29] Kapinova A. Stefanicka P. Kubatka P. Zubor P. Uramova S. Kello M. (2017). Are Plant-Based Functional Foods Better Choice against Cancer Than Single Phytochemicals? A Critical Review of Current Breast Cancer Research. Biomed. Pharmacother. 96, 1465–1477. 10.1016/j.biopha.2017.11.134 29198744

[B30] Kaushik N. Yang H. Jeong S. Kaushik N. K. Bhartiya P. Nhat Nguyen L. (2020). Antiproliferative Activity of Pyracantha and Paullinia Plant Extracts on Aggressive Breast and Hepatocellular Carcinoma Cells. Appl. Sci. 10, 7543. 10.3390/app10217543

[B31] Kim C. R. Choi S. J. Kwon Y. K. Kim J. K. Kim Y.-J. Park G. G. (2016a). *Cinnamomum loureirii* Extract Inhibits Acetylcholinesterase Activity and Ameliorates Trimethyltin-Induced Cognitive Dysfunction in Mice. Biol. Pharm. Bull. 39, 1130–1136. 10.1248/bpb.b16-00045 27374288

[B32] Kim J. H. Choi Y. B. Lee H. J. Kim Y. H. Kim J. H. Sim J. M. (2016b). Fourier Transform Ion Cyclotron Resonance (FT-ICR) MASS Spectrophotometric Analysis of Flower Petal From Paeonia Lactiflora Cv. 'Red Charm' and Evaluation of its Functional Activity. Korean J. Plant Resour. 29, 588–597. 10.7732/kjpr.2016.29.5.588

[B33] Kim M.-J. Ryu G. R. Kang J.-H. Sim S. S. Min D. S. Rhie D.-J. (2004). Inhibitory Effects of Epicatechin on Interleukin-1β-Induced Inducible Nitric Oxide Synthase Expression in RINm5F Cells and Rat Pancreatic Islets by Down-Regulation of NF-Κb Activation. Biochem. Pharmacol. 68, 1775–1785. 10.1016/j.bcp.2004.06.031 15450943

[B34] Kubatka P. Kello M. Kajo K. Kruzliak P. Výbohová D. Mojžiš J. (2017). Oregano Demonstrates Distinct Tumour-Suppressive Effects in the Breast Carcinoma Model. Eur. J. Nutr. 56, 1303–1316. 10.1007/s00394-016-1181-5 26907089

[B35] Kubatka P. Kello M. Kajo K. Kruzliak P. Výbohová D. Šmejkal K. (2016). Young Barley Indicates Antitumor Effects in Experimental Breast Cancer *In Vivo* and *In Vitro* . Nutr. Cancer. 68, 611–621. 10.1080/01635581.2016.1154577 27042893

[B36] Kumari S. Kumari P. Panda P. K. Pramanik N. Verma S. K. Mallick M. A. (2019). Molecular Aspect of Phytofabrication of Gold Nanoparticle from Andrographis Peniculata Photosystem II and Their *In Vivo* Biological Effect on Embryonic Zebrafish (*Danio rerio*). Environ. Nanotechnology, Monit. Management. 11, 100201. 10.1016/j.enmm.2018.100201

[B37] Kurokawa M. Kumeda C. A. Yamamura J.-I. Kamiyama T. Shiraki K. (1998). Antipyretic Activity of Cinnamyl Derivatives and Related Compounds in Influenza Virus-Infected Mice. Eur. J. Pharmacol. 348, 45–51. 10.1016/s0014-2999(98)00121-6 9650830

[B38] Kwon H. K. Hwang J. S. So J. S. Lee C. G. Sahoo A. Ryu J. H. (2019). Correction to: Cinnamon Extract Induces Tumor Cell Death Through Inhibition of NFκB and AP1. BMC Cancer. 19, 1113. 10.1186/s12885-019-6342-5 31727003 PMC6857335

[B39] Kwon S. W. Hong S. S. Kim J. I. Ahn I. H. (2003). Antioxidant Properties of Heat-Treated *Hibiscus Syriacus* . Biol. Bull. 30, 15–16. 10.1023/A:1022055224858 12647536

[B40] Li R. Wang Y. Jiang Z.-T. Jiang S. (2010). Chemical Composition of the Essential Oils of*Cinnamomum loureirii* Nees. From China Obtained by Hydrodistillation and Microwave-Assisted Hydrodistillation. J. Essent. Oil Res. 22, 129–131. 10.1080/10412905.2010.9700281

[B41] Maganha E. G. Halmenschlager R. d. C. Rosa R. M. Henriques J. A. P. Ramos A. L. L. d. P. Saffi J. (2010). Pharmacological Evidences for the Extracts and Secondary Metabolites From Plants of the Genus Hibiscus. Food Chem. 118, 1–10. 10.1016/j.foodchem.2009.04.005

[B42] Mann J. (2002). Natural Products in Cancer Chemotherapy: Past, Present and Future. Nat. Rev. Cancer 2, 143–148. 10.1038/nrc723 12635177

[B43] Mathew S. Abraham T. E. (2006). Studies on the Antioxidant Activities of Cinnamon (*Cinnamomum verum*) Bark Extracts, through Various *In Vitro* Models. Food Chem. 94, 520–528. 10.1016/j.foodchem.2004.11.043

[B44] Mishra A. P. Saklani S. Salehi B. Parcha V. Sharifi-Rad M. Milella L. (2018). Satyrium Nepalense, a High Altitude Medicinal Orchid of Indian Himalayan Region: Chemical Profile and Biological Activities of Tuber Extracts. Cell Mol Biol (Noisy-le-grand). 64, 35–43. 10.14715/cmb/2018.64.8.6 29981681

[B45] Navya P. N. Kaphle A. Srinivas S. P. Bhargava S. K. Rotello V. M. Daima H. K. (2019). Current Trends and Challenges in Cancer Management and Therapy Using Designer Nanomaterials. Nano Converg. 6, 23. 10.1186/s40580-019-0193-2 31304563 PMC6626766

[B46] Nguyen L. N. Kaushik N. Bhartiya P. Gurmessa S. K. Kim H.-J. Nguyen L. Q. (2021). Plasma-Synthesized Mussel-Inspired Gold Nanoparticles Promote Autophagy-Dependent Damage-Associated Molecular Pattern Release to Potentiate Immunogenic Cancer Cell Death. J. Ind. Eng. Chem. 100, 99–111. 10.1016/j.jiec.2021.05.035

[B47] Ooi L. S. M. Li Y. Kam S.-L. Wang H. Wong E. Y. L. Ooi V. E. C. (2012). Antimicrobial Activities of Cinnamon Oil and Cinnamaldehyde From the Chinese Medicinal Herb*Cinnamomum cassia* Blume. Am. J. Chin. Med. 34, 511–522. 10.1142/s0192415x06004041 16710900

[B48] Pietta P.-G. (2000). Flavonoids as Antioxidants. J. Nat. Prod. 63, 1035–1042. 10.1021/np9904509 10924197

[B49] Pistollato F. Giampieri F. Battino M. (2015). The Use of Plant-Derived Bioactive Compounds to Target Cancer Stem Cells and Modulate Tumor Microenvironment. Food Chem. Toxicol. 75, 58–70. 10.1016/j.fct.2014.11.004 25445513

[B50] Rana V. S. Blazquez M. A. (2007). Chemical Constituents of Gynura CusimbuaAerial Parts. J. Essent. Oil Res. 19, 21–22. 10.1080/10412905.2007.9699219

[B51] Rawal S. Patel M. M. (2019). Threatening Cancer With Nanoparticle Aided Combination Oncotherapy. J. Controlled Release. 301, 76–109. 10.1016/j.jconrel.2019.03.015 30890445

[B52] Reveneau S. Arnould L. Jolimoy G. Hilpert S. Lejeune P. Saint-Giorgio V. (1999). Nitric Oxide Synthase in Human Breast Cancer Is Associated With Tumor Grade, Proliferation Rate, and Expression of Progesterone Receptors. Lab. Invest. 79, 1215–1225. 10532585

[B53] Sarikurkcu C. Cengiz M. Uren M. C. Ceylan O. Orenc T. Tepe B. (2016). Phenolic Composition, Enzyme Inhibitory, and Antioxidant Activity of Bituminaria Bituminosa. Food Sci. Biotechnol. 25, 1299–1304. 10.1007/s10068-016-0204-6 30263408 PMC6049266

[B54] Schoene N. W. Kelly M. A. Polansky M. M. Anderson R. A. (2005). Water-Soluble Polymeric Polyphenols From Cinnamon Inhibit Proliferation and Alter Cell Cycle Distribution Patterns of Hematologic Tumor Cell Lines. Cancer Lett. 230, 134–140. 10.1016/j.canlet.2004.12.039 16253769

[B55] Selim Y. A. Azb M. A. Ragab I. H. M. Abd El-Azim M. M. (2020). Green Synthesis of Zinc Oxide Nanoparticles Using Aqueous Extract of Deverra Tortuosa and Their Cytotoxic Activities. Sci. Rep. 10, 3445. 10.1038/s41598-020-60541-1 32103090 PMC7044426

[B56] Sharifi-Rad M. Nazaruk J. Polito L. Morais-Braga M. F. B. Rocha J. E. Coutinho H. D. M. (2018). Matricaria Genus as a Source of Antimicrobial Agents: From Farm to Pharmacy and Food Applications. Microbiol. Res. 215, 76–88. 10.1016/j.micres.2018.06.010 30172312

[B57] Sheel R. Kumari P. Panda P. K. Jawed Ansari M. D. Patel P. Singh S. (2020). Molecular Intrinsic Proximal Interaction Infer Oxidative Stress and Apoptosis Modulated *In Vivo* Biocompatibility of P.Niruri Contrived Antibacterial Iron Oxide Nanoparticles with Zebrafish. Environ. Pollut. 267, 115482. 10.1016/j.envpol.2020.115482 32889517

[B58] Sicińska P. Mokra K. Wozniak K. Michałowicz J. Bukowska B. (2021). Genotoxic Risk Assessment and Mechanism of DNA Damage Induced by Phthalates and Their Metabolites in Human Peripheral Blood Mononuclear Cells. Sci. Rep. 11, 1658. 10.1038/s41598-020-79932-5 33462290 PMC7814068

[B59] Szejk M. Kołodziejczyk-Czepas J. Żbikowska H. M. (2016). Radioprotectors in Radiotherapy - Advances in the Potential Application of Phytochemicals. Postepy Hig Med. Dosw. 70, 722–734. 10.5604/17322693.1208039 27356603

[B60] Takanashi K. Suda M. Matsumoto K. Ishihara C. Toda K. Kawaguchi K. (2017). Epicatechin Oligomers Longer Than Trimers Have Anti-Cancer Activities, but Not the Catechin Counterparts. Sci. Rep. 7, 7791. 10.1038/s41598-017-08059-x 28798415 PMC5552761

[B61] Verma M. L. Rani V. (2021). Biosensors for Toxic Metals, Polychlorinated Biphenyls, Biological Oxygen Demand, Endocrine Disruptors, Hormones, Dioxin, Phenolic and Organophosphorus Compounds: a Review. Environ. Chem. Lett. 19, 1657–1666. 10.1007/s10311-020-01116-4

[B62] Verma S. K. Jha E. Kiran K. J. Bhat S. Suar M. Mohanty P. S. (2016). Synthesis and Characterization of Novel Polymer-Hybrid Silver Nanoparticles and its Biomedical Study. Mater. Today Proc. 3, 1949–1957. 10.1016/j.matpr.2016.04.096

[B63] Wen T.-C. Li Y.-S. Rajamani K. Harn H.-J. Lin S.-Z. Chiou T.-W. (2018). Effect of *Cinnamomum* Osmophloeum Kanehira Leaf Aqueous Extract on Dermal Papilla Cell Proliferation and Hair Growth. Cell Transpl. 27, 256–263. 10.1177/0963689717741139 PMC5898689 29637818

[B64] Wink D. A. Vodovotz Y. Cook J. A. Krishna M. C. Kim S. Coffin D. (1998). The Role of Nitric Oxide Chemistry in Cancer Treatment. Biochemistry (Mosc). 63, 802–809. 9721332

[B65] Xu X. Y. Tran T. H. M. Perumalsamy H. Sanjeevram D. Kim Y.-J. (2021). Biosynthetic Gold Nanoparticles of Hibiscus Syriacus L. Callus Potentiates Anti-Inflammation Efficacy via an Autophagy-Dependent Mechanism. Mater. Sci. Eng. C. 124, 112035. 10.1016/j.msec.2021.112035 33947536

[B66] Yang X. Q. Zheng H. Ye Q. Li R. Y. Chen Y. (2015). Essential Oil of Cinnamon Exerts Anti-Cancer Activity Against Head and Neck Squamous Cell Carcinoma via Attenuating Epidermal Growth Factor Receptor - Tyrosine Kinase. J. BUON. 20, 1518–1525. 26854449

[B67] Yuan H. J. Li W. Jin J. M. Chen J. J. Jiang J. Wang H. (2017). Research Progress on Chemical Constituents, Pharmacological Mechanism and Clinical Application of Guizhi Decoction. Zhongguo Zhong Yao Za Zhi. 42, 4556–4564. 10.19540/j.cnki.cjcmm.20170928.010 29376252

[B68] Zengin G. Sarikurkcu C. Aktumsek A. Ceylan R. Ceylan O. (2014). A Comprehensive Study on Phytochemical Characterization of Haplophyllum Myrtifolium Boiss. Endemic to Turkey and its Inhibitory Potential Against Key Enzymes Involved in Alzheimer, Skin Diseases and Type II Diabetes. Ind. Crops Prod. 53, 244–251. 10.1016/j.indcrop.2013.12.043

[B69] Zhu Z. P. Zhang M. F. Shen Y. Q. Chen G. J. (1993). Pharmacological Study on Spleen-Stomach Warming and Analgesic Action of *Cinnamomum cassia* Presl. Zhongguo Zhong Yao Za Zhi. 18, 553–557. 8011112

